# The Influence of IS*1301* in the Capsule Biosynthesis Locus on Meningococcal Carriage and Disease

**DOI:** 10.1371/journal.pone.0009413

**Published:** 2010-02-25

**Authors:** Elisabeth Kugelberg, Bridget Gollan, Christopher Farrance, Holly Bratcher, Jay Lucidarme, Ana Belén Ibarz-Pavón, Martin C. J. Maiden, Ray Borrow, Christoph M. Tang

**Affiliations:** 1 Centre for Molecular Microbiology and Infection, Department of Microbiology, Imperial College London, London, United Kingdom; 2 Department of Zoology, Oxford University, Oxford, United Kingdom; 3 Manchester Royal Infirmary, Health Protection Agency, Manchester, United Kingdom; University of Florida, United States of America

## Abstract

Previously we have shown that insertion of IS*1301* in the *sia*/*ctr* intergenic region (IGR) of serogroup C *Neisseria meningitidis* (MenC) isolates from Spain confers increased resistance against complement-mediated killing. Here we investigate the significance of IS*1301* in the same location in *N. meningitidis* isolates from the UK. PCR and sequencing was used to screen a collection of more than 1500 meningococcal carriage and disease isolates from the UK for the presence of IS*1301* in the IGR. IS*1301* was not identified in the IGR among vaccine failure strains but was frequently found in serogroup B isolates (MenB) from clonal complex 269 (cc269). Almost all IS*1301* insertions in cc269 were associated with novel polymorphisms, and did not change capsule expression or resistance to human complement. After excluding sequence types (STs) distant from the central genotype within cc269, there was no significant difference for the presence of IS*1301* in the IGR of carriage isolates compared to disease isolates. Isolates with insertion of IS*1301* in the IGR are not responsible for MenC disease in UK vaccine failures. Novel polymorphisms associated with IS*1301* in the IGR of UK MenB isolates do not lead to the resistance phenotype seen for IS*1301* in the IGR of MenC isolates.

## Introduction

The exclusively human pathogen *Neisseria meningitidis* causes serious diseases including septicaemia and bacterial meningitis. However, it is primarily a commensal of the human nasopharynx in around 10% of healthy individuals [1], [2]. Rarely, the bacterium overcomes host defences, especially the complement system, and causes bloodstream infection, septic shock and/or meningitis. The meningococcus can express one of 13 capsules (classifying isolates into serogroups) that are necessary for avoidance of complement-mediated killing [3]. Isolates are also categorised into sequence types (STs) based on sequences of seven housekeeping genes [4]. Most disease is caused by a limited number of genotypes, while other genotypes seldom invade the bloodstream [5]. Clonal complexes (cc) are groupings of STs with similar allelic profiles typically sharing at least 4–5 loci (http://pubmlst.org/neisseria).

**Figure 1:**
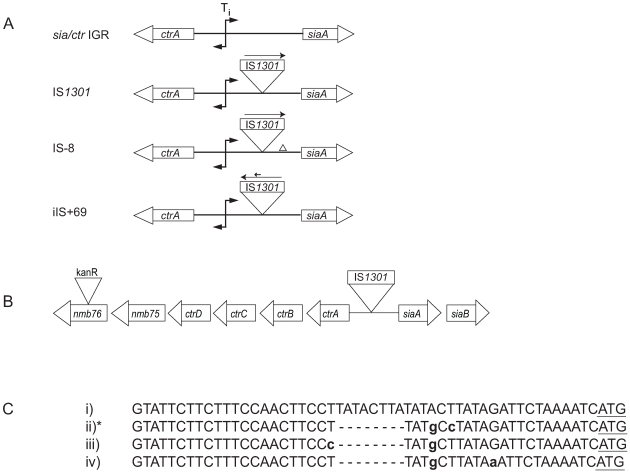
The intergenic region (IGR) between the operons for capsule transport (*ctr*) and biosynthesis (*sia*). A: The transcription initiation site (Ti) is indicated [28] as well as the insertion of IS*1301* in the IGR. The two most common polymorphic IGR with IS*1301*, IS-8 and iIS+69, are also illustrated. B: Isogenic strains with and without the polymorphisms associated with IS*1301* were constructed by tagging the *nmb76* gene downstream of the *ctr* operon with a kanamycin resistance (kan^R^) cassette. C: The 8 bp deletion associated with IS*1301* in the IGR; i) wild-type sequence, and ii) to iv) shows the 8 bp deletion with associated base pair changes (indicated in bold and lower case). * Refers to the most common type of IS-8, which was used for phenotypic analysis. The sequence denotes the base pairs from the insertion site of IS*1301* up to the *siaA* translational start (underlined).

Recently we described a polymorphism in three serogroup C ST-11 *N. meningitidis* isolates (MenC) from Spain that enhances resistance against complement-mediated killing, the principal mechanism of immunity against meningococcal disease [6]. In all isolates, IS*1301* was found in the 134 bp intergenic region (IGR) between operons for capsule biosynthesis (*sia*) and export (*ctr*) (Figure 1A), leading to up-regulation of capsule expression and providing a generic mechanism for increased resistance against bactericidal antibodies.

The MenC glycoconjugate vaccine has been very effective, leading to >90% reduction of MenC cases in countries where it has been introduced [7]–[10]. So far there has been no evidence of emergence of strains that are resistant to the immune responses elicited by the vaccine. However, there are a few reports of MenC disease developing in subjects who have completed a course of immunisation [11], [12], and these could be caused by strains with increased resistance to complement-mediated killing due to insertion of IS*1301* in the *sia*/*ctr* IGR. Furthermore, increased resistance to complement-mediated killing conferred by IS*1301* could promote meningococcal virulence. Here we investigate whether isolates with IS*1301* in the IGR are emerging among vaccine failure strains, and compare its presence in UK carriage and disease isolates.

## Results

### IS*1301* in the IGR Is Not Associated with MenC Conjugate Vaccine Failure in the UK

IS*1301* in the *sia*/*ctr* IGR was originally described in MenC isolates in Spain; isolates had low titre (<8) in serum bactericidal assays (SBA) using sera from individuals immunised with the MenC conjugate vaccine [6]. As an SBA titre of <8 is a marker of protective immunity [13], [14], we examined MenC isolates from individuals from the UK with meningococcal disease who had completed a course of the MenC conjugate vaccine [11], [15]. Most isolates were ST-11 (79%; 26 out of 33, with the others were ST-1095 [two isolates], ST-8, ST-2709, ST-467, ST-3463 and ST-6331), the most common cause of MenC disease at the time of the introduction of the vaccine [16]. IS*1301* was not present in the IGR of any of the 33 MenC isolates, even though most isolates (32 out of 33) have IS*1301* elsewhere in the genome (data not shown). We also examined MenC disease isolates in the UK before the introduction of the MenC conjugate vaccine (1998–99), and found IS*1301* in the IGR in less than 1% of the isolates (1 out of 104 isolates, Table 1); IS*1301* was inserted at the same location but in the opposite orientation (inverted) to the ST-11 isolates from Spain [6]. Therefore, there is currently no evidence from the UK that IS*1301* in the IGR mediates a mechanism for escape from the MenC conjugate vaccine.

**Table 1:** Presence of IS*1301* in the IGR of UK disease and carriage isolates from South East England.

Serogroup	Disease		Carriage		P-value^a^
	Total	+ IS*1301*	Total	+ IS*1301*	
B	209	30 (14%)	175	11 (6%)	0.01
C	104	1 (<1%)	16	0	
W135	15	0	53	0	
Y	5	0	46	0	
A	1	0	0	0	
X	1	0	10	0	
Z	0	0	6	0	
29E	1	0	70	0	
NG^b^	9	2	365	0	
Total	345	33 (10%)	741	11 (1%)	<0.0001

a P-value was calculated using Fischer's exact test.

b NG, non-groupable.

### Presence of IS*1301* in the IGR of UK Meningococcal Disease and Carriage Isolates

Enhanced resistance to human sera may promote the virulence of *N. meningitidis* during infection, and therefore insertion of IS*1301* in the IGR might be more frequent among meningococcal disease compared to carriage isolates. We examined 741 carriage and 345 disease isolates of different serogroups collected between 1998 and 2000 in the South East of England (Table 1, [17], [18]). In this collection, insertion of IS*1301* was significantly more frequent in the IGR of disease (10%; 33 out of 345) compared to carriage isolates (1.5%; 11 out of 741, p<0.0001, Table 1). Approximately 50% of the carriage isolates were unencapsulated (non-groupable), and the presence/absence of IS*1301* in the IGR should not affect the phenotype of these isolates. To exclude this as a potential confounding factor, we restricted our analysis to serogroupable isolates. There was still a significant difference for the presence of IS*1301* in the IGR in disease (31 out of 336) compared to carriage isolates (11 out of 365, p = 0.0004) even after excluding unencapsulated isolates.

Insertion of IS*1301* was most frequently found in MenB isolates belonging to clonal complex ST-269 (cc269) with 24 of 55 having IS*1301* in the IGR (Table 2). Cc269 causes a significant proportion of meningococcal cases in UK [16], [19], and in the collection, cc269 isolates were more commonly found among disease isolates (16%; 55 out of 345) compared to carriage isolates (3%; 22 out of 741, p<0.0001). To account for this potential bias, we analysed only cc269 isolates, and still found a significant difference between the presence of IS*1301* in the IGR of disease isolates (44%; 24 out of 55) compared to carriage isolates (14%; 3 out of 22, p = 0.017).

**Table 2:** Presence of IS1301 in the IGR of MenB and MenC isolates from different clonal complexes.

Clonal complex	MenB Disease		MenB Carriage		MenC Disease		MenC Carriage	
	Total	+IS1301	Total	+IS1301	Total	+IS1301	Total	+IS1301
ST-41/44 complex/Lineage 3	94	1 (1%)	67	0	2	0	1	0
ST-269	55	24 (44%)	22	3 (14%)	2	0	2	0
ST-32 complex	18	2 (11%)	5	0	1	0	0	0
ST-11 complex	2	0	0	0	85	0	8	0
ST-35	3	0	16	0	0	0	1	0
ST-461	3	0	1	0	0	0	0	0
ST-103	1	0	1	0	0	0	0	0
ST-1157	1	0	3	0	0	0	0	0
ST-8	2	0	1	0	11	0	1	0
ST-60	9	1 (11%)	3	1 (33%)	0	0	0	0
ST-37	1	0	1	0	0	0	0	0
ST-364	2	0	2	0	0	0	0	0
ST-22	1	0	2	0	0	0	0	0
ST-213	1	0	10	0	0	0	0	0
ST-18	3	0	0	0	2	1 (50%)	0	0
ST-162	1	0	10	0	0	0	0	0
ST-167	1	0	2	0	0	0	0	0
ST-254	0	0	0	0	0	0	1	0
Unassigned	11	1 (9%)	29	7 (24%)	1	0	2	0
Total	209	30 (14%)	175	11 (6%)	104	1 (<1%)	16	0

### IGRs with IS*1301* in MenB Isolates Are Associated with Polymorphisms

Strikingly, only one of 44 IGRs with IS*1301* identified among isolates from South East England was identical to that in Spanish MenC ST-11 isolates (Table 3). Most commonly IS*1301* was associated with an 8 bp deletion upstream of *siaA* (IS-8) or the IGR contained an inverted IS*1301* with an internal 69 bp duplication (iIS+69); these two polymorphisms account for 66% (29 out of 44) of the changes seen. The 8 bp deletion was accompanied by three other sequence changes, one of which represented >80% of IS-8 (type (ii) in Figure 1C) and hence was chosen for further study. The 8 bp deletion is located 14 nucleotides upstream of *siaA* translational start, and has been reported not to influence *siaA* translation [20]. The 69 bp duplication within the inverted IS*1301* duplicates bases 194 to 264 corresponding to nucleotides 580 to 649 of IS*1301* in the original orientation [21].

**Table 3:** Characteristics of IGRs with IS*1301* in 44 MenB isolates from South East England.

	IS	iIS	IS-8	iIS-8	IS+69	iIS+69	IS+72
Disease	1	5	14	4	1	7	1
Carriage	0	2	8	1	0	0	0

Details of IS-8 and iIS+69 are shown in Fig. 1. iIS refers to IS*1301* inserted in the IGR in the opposite orientation and +72 indicates IS*1301* with an additional 72 bp compared with Spanish ST-11 isolates.

### Polymorphic *sia/ctr* IGRs with IS*1301* Lead to Increased Transcription of the Genes for Capsule Transport but Not Capsule Biosynthesis

**Figure 2:**
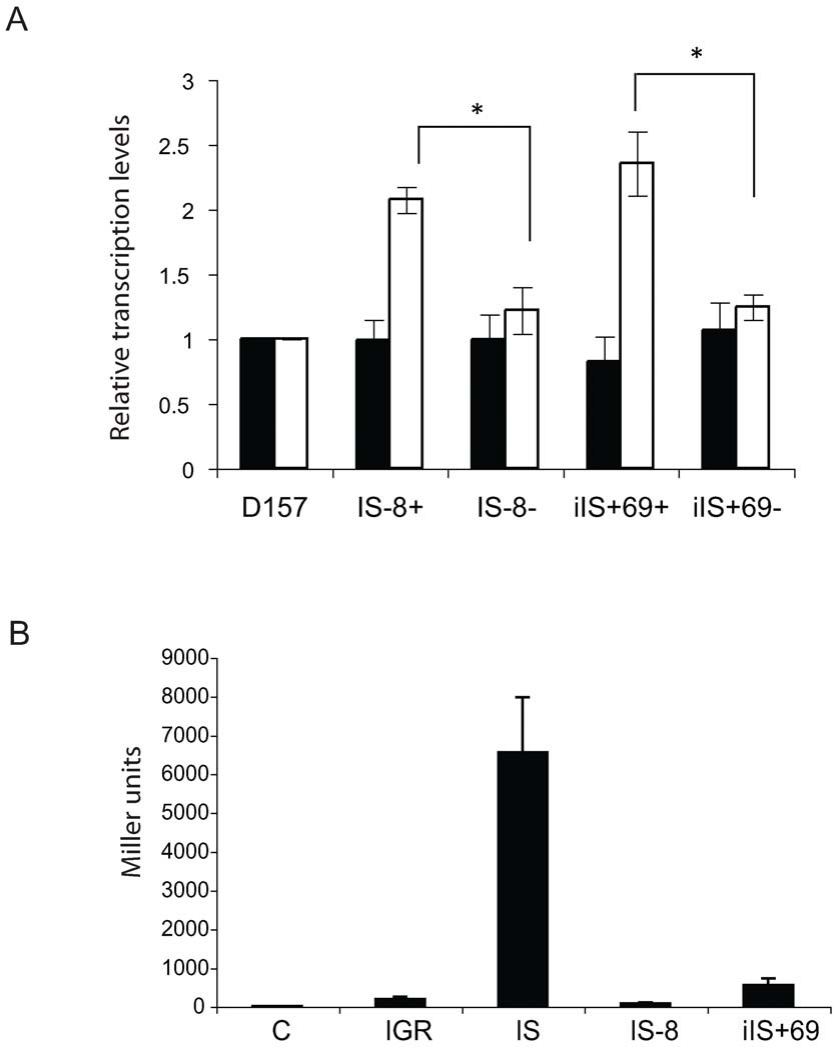
Expression of capsule biosynthesis genes. A: Relative levels of *siaA* (black bars) and *ctrA* (white bars) mRNA transcripts measured by qrtRT-PCR. P<0.005 (*) for differences in *ctrA* mRNA levels in IS-8+ and iIS+69+ versus the controls, using Student's *t* test. B: β-galactosidase activity was determined for *siaA* translational fusions. C denotes a control without an IGR. Results are average of three independent experiments, and error bars indicate the standard deviation (SD).

Insertion of IS*1301* in the *sia*/*ctr* IGR of MenC isolates leads to increased expression of the *sia* and *ctr* operons [6]. To investigate the effect of IS*1301* with associated polymorphisms, we constructed isogenic strains by introducing the two common types of IGRs with IS*1301*, IS-8 and iIS+69, into a MenB cc269 strain. An antibiotic resistance marker was inserted into *nmb76* in strains containing these polymorphisms (Figure 1B), and genomic DNA was used to transform a MenB cc269 disease isolate (D157) without IGR changes; transformants were selected with (IS-8+ and iIS+69+) and without (IS-8- and iIS+69-) changes in the IGR. Compared with the effect of IS*1301* in MenC [6], both IS-8+ and iIS+69+ led to a similar two-fold increase in the expression of *ctrA* as determined by qrtRT-PCR (Figure 2A). However, no upregulation in *siaA* expression was detected in these strains. The expression of *siaA* and *ctrA* in strains without IS*1301* in the IGR was similar to wild-type.

To verify the findings for *siaA* expression obtained by qrtRT-PCR, we constructed *lacZ* translational *sia* fusions in MenB with IGRs without IS*1301*, and with the original IS*1301*, IS-8, or iIS+69. The construct with IS*1301* in the IGR showed a >30-fold increase in reporter activity, whereas fusions with the IGRs found in MenB isolates showed similar levels of β-galactosidase activity as the IGR without changes (Figure 2B), consistent with the qrtRT-PCR results.

### Increased Transcription of Capsule Transport Genes Does Not Change the Amount of Capsule or Resistance against Complement-Mediated Killing

**Figure 3:**
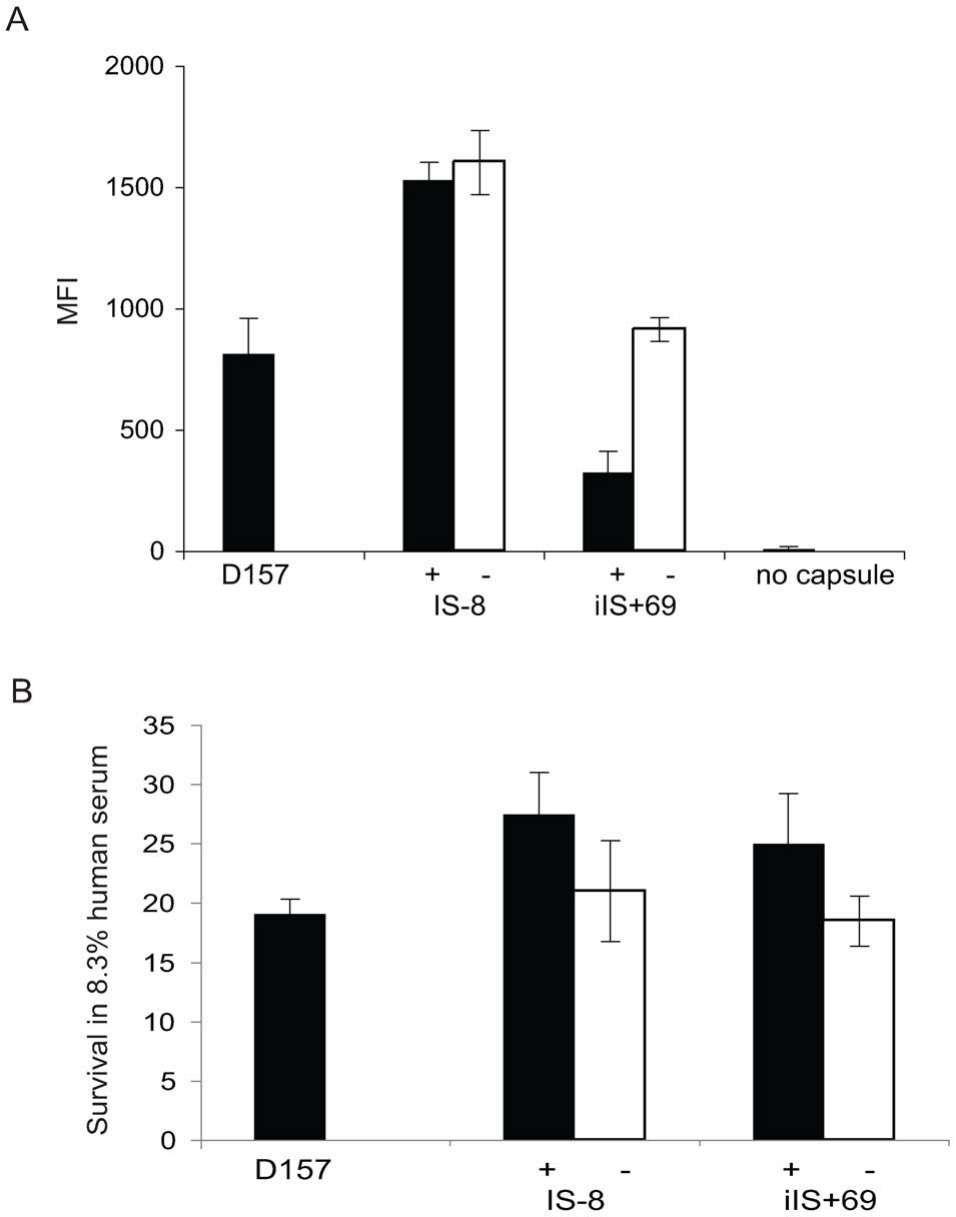
Polymorphic IGRs do not influence capsule expression or complement resistance. A: Relative amount of capsule detected by FACS. Results are shown as mean fluorescense index (MFI; calculated as the geometric mean multiplied by the percentage of positive cells). B: Survival in 8.33% human serum after 1 hour incubation at 37(C. Experiments were performed in duplicate on at least three independent occasions, and error bars indicate the SD. White bars represent isogenic control strains without IS*1301* in the IGR.

It is not known whether increased expression of only capsule transport genes is sufficient to increase capsule expression. FACS analysis of the amount of capsule expressed by the strains IS-8+, IS-8–, iIS+69+, iIS+69–, and D157 showed that strains with polymorphic IGRs with IS*1301* do not express increased amounts of capsule compared to isogenic strains without the polymorphic IGRs (Figure 3A). Although there was no detectable difference in the amount of capsule, the novel polymorphic IGRs with IS*1301* could still influence resistance against complement-mediated killing. However, SBA titres were similar for strains with and without the polymorphic IGRs with IS*1301* (50% killing of all strains was seen at a 1/512 dilution of human immune sera, not shown). This was confirmed by human serum assays, in which there was no difference in the survival of isogenic strains with and without IS*1301* with associated polymorphisms (Figure 3B).

### Presence of IS*1301* in MenB cc269 Carriage and Disease Isolates

Phenotypic analyses suggest that MenB IGR polymorphisms might have no or little impact on meningococcal pathogenesis. This is in contrast to our observation that the polymorphic IGRs with IS*1301* were more frequent in disease compared to carriage isolates in the survey from South East England. IS*1301* was predominantly found among MenB cc269 isolates, of which only a limited number were examined (55 disease and 22 carriage isolates). We examined a larger number of MenB cc269 with 241 carriage isolates and 421 disease isolates from seven regions of the UK. Consistent with the South East England isolates, insertion of IS*1301* in the IGR was significantly more frequent among disease isolates (35%; 148 out of 421) compared to carriage isolates (26%; 63 out of 241, p<0.05). All IS*1301* insertions in cc269 were polymorphic and the majority (>80%; 178 out of 211) were IS-8 or iIS+69. Again when excluding non-groupable carriage isolates from the analysis, there was still a significant difference between the presence of IS*1301* in the IGR of disease isolates (35%) compared to carriage isolates (26%; 49 out of 187, p<0.05).

**Figure 4:**
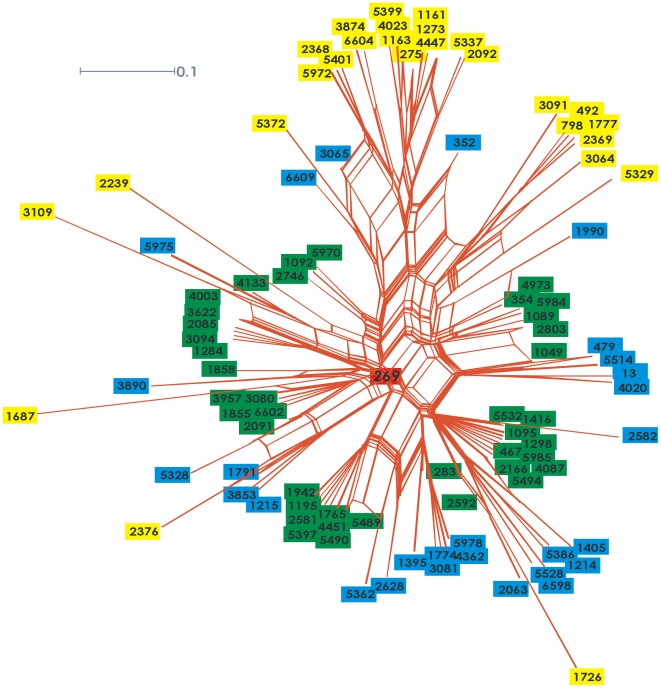
Genetic relatedness between STs within cc269. Neighbor-net constructed from the concatenation of the seven MLST housekeeping genes of *N. meningitidis* [4]. The major groupings (indicated with green, black, and yellow circles) represent the number of allelic differences (1, 2 and 3 respectively) from the core ST-269 allelic profile. The tree was made using the Split-Tree4 software [29].

**Figure 5:**
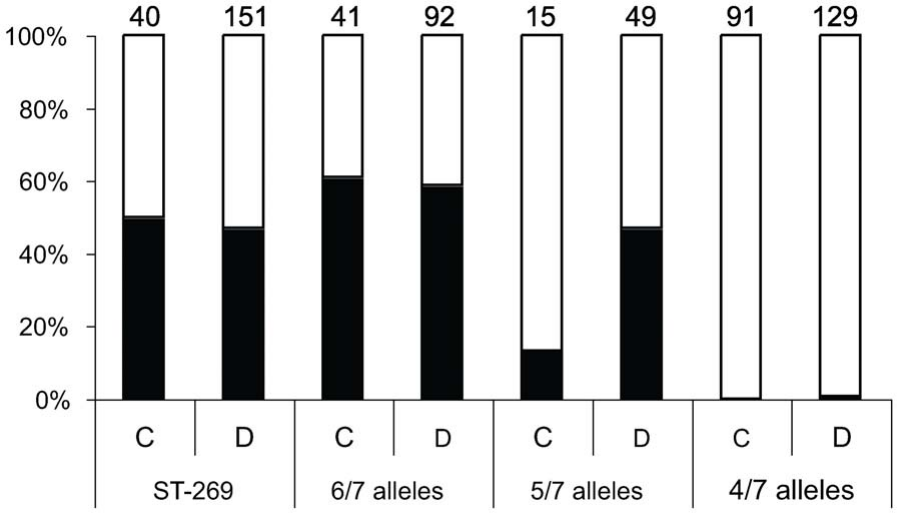
Presence of IS*1301* in the IGR of carriage isolates and disease isolates. Carriage (C) and disease (D) isolates were analysed for the presence of IS*1301* in the IGR depending on the numbers of loci the STs share with ST-269. Black bars represent strains with IS*1301* in the IGR and white bars show the frequency of isolates without IS*1301* in the IGR. Numbers above bars indicate the total number of isolates.

Next we extended our analysis to examine potential variation within cc269. Some STs in the clonal complex are relatively distant from the ancestral ST-269 and share only four out of seven MLST loci with ST-269 (Figure 4); most of these STs (e.g. 1161 and 1163) cluster together around the subgroup-founder ST-275. IS*1301* in the IGR, or indeed anywhere in the genome, is rare among these STs, present in only one out of 217 isolates (Figure 5). Isolates which share four out of seven loci with ST-269 are significantly more prevalent in carriage (48%, 91 of 187) compared with disease isolates (31%, 129 of 421). This is distinct from other isolates in cc269 (that share five or more loci with ST-269) of which approximately 50% have IS*1301* in the IGR, with a similar prevalence among carriage and disease isolates (Figure 5). Therefore we excluded from our analysis all STs sharing only four out of seven loci with ST-269. Strikingly, there was no longer a significant difference between the presence of IS*1301* in the IGR of disease (51%; 148 out of 292) compared to carriage isolates (49%; 47 out of 96, p = 0.80).

## Discussion

The presence of IS*1301* in the *sia*/*ctr* IGR of MenC ST-11 enhances capsule expression and resistance against bactericidal antibodies elicited by the MenC conjugate vaccine [6]. Although the MenC vaccine has been successfully introduced across Europe (with >90% efficacy [8], [10]), a small number of vaccinated individuals still develop MenC disease [11], [12]. As isolates with IS*1301* in the IGR are resistant to complement, it was possible that they might lead to such vaccine failure. However, MenC isolates from people immunised with the MenC vaccine in the UK had no evidence of IS*1301* in the IGR. It is possible that these isolates have other polymorphisms that contribute to their ability to be virulent in the vaccinees, or that the cases have arisen through waning host immunity rather than bacterial factors [11].

Our initial study of over 1,000 isolates from South East England showed a highly significant association of IS*1301* in the IGR among disease versus carriage isolates. Closer examination of the dataset showed that IS*1301* in the IGR was largely restricted to certain isolates, especially those in cc269. This is an important clonal complex, which accounts for a substantial proportion of UK disease cases [16], [19], [22]. Therefore, we examined the frequency of the IS*1301* in the IGR in a larger collection of cc269 carriage (n = 241) and disease (n = 421) isolates across England and Wales, and still observed a significant association of IS*1301* in the IGR in disease compared to carriage isolates.

Closer inspection of the IGRs with IS*1301* in MenB revealed that they are associated with novel polymorphisms. The most frequent polymorphisms, IS-8 and iIS+69 (which account for >80% of changes), abrogate the up-regulation of capsule expression associated with insertion of IS*1301* in MenC and do not confer increased resistance to complement-mediated killing. Nearly all IS*1301* insertions in the original orientation [6] (99%) identified in this study were associated with an 8 bp deletion upstream of the *siaA* start. This indicates that this small deletion influences the functional consequences of IS*1301* insertion, even though, on its own, it does not change transcriptional activity [20]. IS*1301* is predicted to have two open reading frames (ORFs) [21]. It is not clear whether the 69 bp internal duplication (which is in the second ORF of IS*1301*) or the orientation of the insertion sequence is responsible for the lack of *sia* up-regulation in isolates containing the iIS+69 polymorphism.

The mechanisms by which IS*1301* insertion leads to up-regulation of *sia* and *ctr* expression are not understood. Indeed, surprisingly little is known about the genetic regulation of this key virulence determinant, or the transcription factors that govern expression. IS*1301* could provide novel transcriptional start sites, prevent binding of transcription factors, or change their interaction with RNA polymerase. It is therefore interesting that both iIS+69 and IS-8 lead to upregulation of *ctr* expression as in the ST-11 isolates. This occurs regardless of the orientation of IS*1301* or its precise sequence, suggesting that increased *ctr* transcription upon IS*1301* insertion may result from changes in spacing due to the additional sequence in the promoter. However, *sia* expression was not elevated in either iIS+69 or in IS-8. The reasons for this are unknown and are under further investigation.

The association of polymorphisms with IS*1301* in cc269 MenB suggests that increased expression of capsule might lead to a significant loss of fitness in these strains, in contrast to MenC ST-11 isolates. The reason for this apparent difference between the two groups of isolates is not obvious. However, it is interesting to note that isolates belonging to ST-11 are rarely found in carriage studies, and when they are present in the nasopharynx, are more likely to express a polysaccharide capsule than other carriage isolates [14].

Isolates from certain STs such as 275, 1161 and 1163 were remarkable as less than 2% have IS*1301* in the IGR or anywhere in their genome. While these isolates have been assigned to cc269, they only share four out of seven MLST loci with ST-269 and appear to constitute a divergent cluster, centred around ST-275. Presence of IS*1301* in the genome and sequence comparison of vaccine candidates among cc269 isolates supports that these isolates form or are part of a clonal complex that is distinct from cc269 [22].

Attempts are being made to identify virulence determinants by comparing the frequency of genetic traits between disease and carriage isolates [23], [24]. In this study we were only alerted to potential confounding effects by the lack of increased complement resistance conferred by the common IGR changes found in MenB isolates. This demonstrates that even minor associated polymorphisms or sequence variation within genetic traits might have a major influence on their effect. Our findings also indicate that meaningful epidemiological comparisons of disease and carriage populations can only be obtained by matching for individual STs, and not at the level of clonal complex, especially when the traits being examined do not confer an observable phenotype. This is a major challenge, as it requires extensive collections that include sufficient numbers of relevant STs. Such large-scale prospective studies are needed to serve as a valuable resource.

## Materials and Methods

### Bacterial Strains and Growth Conditions

MenC conjugate vaccine failure strains were recovered from 33 patients in the UK who had completed a course of immunisation yet contracted meningococcal disease [11]. All disease isolates were obtained from sterile sites of patients from the UK with invasive meningococcal disease from 1998 to 2007. Carriage isolates were collected in November and December in 1999 and 2001 among 15- to 19-year old students in seven centres in the UK: Bangor, Cardiff, Glasgow, Nottingham, Plymouth, Stockport and Oxfordshire [18], [25]. A MenB ST-269 disease isolate without changes in the IGR, D157 (Meningococcal Reference Unit number M01.241107), was used for construction of isogenic strains. *Escherichia coli* was used for construction of the *nmb76* mutant and translational fusions.

*N. meningitidis* was grown on Brain Heart Infusion (BHI) medium with 5% Levanthal's supplement at 37°C in 5% CO2; kanamycin (kan) was used at 100 µg/ml and erythromycin at 2 µg/ml. *E. coli* was grown on Luria-Bertani (LB) broth with 50 µg/ml of kan or 200 µg/ml of erythromycin, as required.

### Molecular Methods

The presence of IS1301 in the IGR and the genome was detected as described previously [6]. Primers used for PCR of the IGR were used for DNA sequencing. To make isogenic strains, isolates were tagged with an antibiotic marker as shown in Figure 1B. Nmb76 was inactivated by *in vitro* Tn*5* transposition. Primers NG1494-for with a *Bam*HI site (5′-CTATGGGATCCGATTTTGACTTATGCACACACCA-3′), and NG1495-rev with an *Nhe*I site (5′-GATCGCTAGCGGAAGCTATTTTCTCTTTATTACCG-3′) were used to amplify nmb76 from MC58. The PCR product was purified, digested with *Bam*HI and *Nhe*I, and ligated into pTrcHis (Invitrogen) lacking a kan resistance cassette. The resulting vector was subjected to *in vitro* mutagenesis (EPICENTRE); PCR and DNA sequencing verified insertion of Tn*5* at nucleotide 722 of *nmb76*. The nmb76 construct was transformed into D157 (generating D157*nmb76*), a strain (M99.240027) with an intact IS*1301* in the IGR with an 8 bp deletion (IS-8), and a strain (M99.240335) with an inverted IS*1301* with an internal 69 bp duplication (iIS+69, Figure 1AB). Genomic DNA from the tagged strains was used to transform D157 to kan resistance. Depending on the site of the cross-overs, transformants did (IS-8+ and iIS+69+) or did not (IS-8– and iIS+69–) contain IS*1301* in the IGR.

For RNA isolation, bacteria were grown in liquid BHI to mid-logarithmic phase and RNA was extracted from 2×10^8^ cells with the RNeasy kit (QIAGEN). Transcript levels of *ctrA* and *siaA* were compared to levels of *gdh* [6]. Results were analysed by comparative quantitation (Rotor-Gene Analysis Software, version 6.0, Corbett Research). RNA was isolated on two different occasions and transcript levels determined twice for each sample.

### SiaA Translational Fusions and β-Galactosidase Aassays

LacZ translational fusions were constructed in pUC19. The erythromycin resistance cassette was amplified from pYHS1882 using NG1304 (5′-GATTAGACGTCCACCGTGTGCTCTACGAC-3′) and NG1305 (5′-GGCAGCCCAGGGGACCATATGTCACAAAAAATAG-3′). The PCR product was digested with *Aat*II and *Eco*O1091 and ligated into pUC19. The construct was digested with *Bsa*I and *Aat*II and ligated with a 589 bp *siaA* fragment (nt. 571 to 1160), amplified using primers NG1306 (5′-GCAGACGTCGCCTAGTGCAAATGGGAGAA-3′) and NG1307 (5′-gcaggtctcatctgtgctggtgcgagtatc). Promoters were amplified with primers NG1324 (5′-TCTGGTAAAGCTTATGCAAAGAATTCT-3′) and NG1419 (5′-GCACATGTCATACGCACACTATTCC-3′) then inserted into the resulting plasmid following digestion with *Hin*dIII and *Afl*III. A construct without an IGR (nt. -597 to -206), amplified with NG1419 and NG1420 (5′-AGAAGCTTTCAG TTATTATATAAGGC-3′), was used as negative control. The *lacZ*α fragment in pUC19 was removed by digestion with *Hin*dIII and *Sfo*I, and replaced with a full-length copy of *lacZ* amplified from pRWX using NG1480 (5′-GGATTCAAGCTTCGTCGTTTTACAAC-3′) and NG1484 (5′-AACCGGGCGCCCAAAAGTTTGTG-3′) [26].

For β-galactosidase assays, 10^9^ cells were inoculated into 10 ml of BHI and grown to mid-logarithmic phase. Cells were centrifuged and washed twice with phosphate buffered saline (PBS). β-galactosidase assays were performed as described previously [27]. Miller units of enzyme activity were calculated using the following equation: Miller Units  = 1000×[(OD_420_−1.75×OD_550_)]/(T×V×OD_600_), where T  =  reaction time (min) and V  =  volume of cells added to the reaction (ml). The values are the average of at least three experiments.

### FACS Analysis

Strains were grown overnight on BHI plates and 2×10^9^ cells were fixed in 3% paraformaldehyde for 1 hr, and washed three times with PBS. Bacteria (2×10^8^ cells) were incubated with 1/10 dilution of serogroup B anticapsular antibody (NIBSC, code 95/750) for 30 min at 37°C. Cells were washed twice in PBS/0.1% Tween 20 (PBS-T), resuspended in PBS containing a FITC-conjugated donkey anti-mouse polyclonal antibody (1∶200 dilution; Jackson Immuno-Research Laboratories), and incubated for 30 min at 4°C in the dark. Samples were washed with PBS-T and fluorescence was measured using an analyzer (FACSCalibur; Becton Dickson), recording at least 10^4^ events. Results were calculated as mean fluorescence index (MFI) [6], which is the geometric mean multiplied by the percentage of positive cells.

Serum bactericidal assay (SBA) and human serum assay. Bacteria were grown overnight on solid medium. For SBAs, 10^4^ cells were incubated for 1 hr at 37°C with serial dilutions of heat-inactivated sera in the presence of 1/12 dilution of baby rabbit complement (Pelfreeze). Cells were plated on solid media and the SBA titres expressed as the reciprocal of the final dilution giving (50% killing compared to controls without complement or without sera. For serum assays, 10^5^ cells were incubated in DMEM with serial dilutions of serum from healthy volunteers for 1 hr at 37°C. Controls were incubated with DMEM media only or with heat-inactivated sera. Survival was calculated by counting the number of bacteria in the input and following incubation in human serum. Significant differences were examined with Student's t test.

### Ethics Statement

Samples were obtained following informed written consent, and procedures were approved by the Riverside Research Ethics Committee (ref. No. 05/Q0401/126).
